# Clearance of Lower Renal Calyceal Residual Fragments Following Lithotripsy in a Patient With Spinal Cord Injury: A Case Report

**DOI:** 10.7759/cureus.94598

**Published:** 2025-10-14

**Authors:** Sergio R Peres Line, Nivaldo S Lavoura, Pedro D Novaes

**Affiliations:** 1 Biosciences, Piracicaba Dental School/University of Campinas, Piracicaba, BRA; 2 Urology, Lavoura Urology Private Clinic, Piracicaba, BRA

**Keywords:** lateral decubitus, lithotripsy, lower-pole stone, renal calculi, residual calculus

## Abstract

The clearance of residual fragments after lithotripsy is a problem of clinical relevance. Residual stone fragments can serve as nucleation centers for the deposition of calcified material, leading to recurrent formation of calculi. This case report presents a 63-year-old male with paraplegia of lower limbs who, through an empirical observation, managed to develop a protocol that resulted in clearance of residual calcified fragments of a lithotripsy performed 267 days ago. Preoperative imaging revealed the presence of kidney stones localized in the lower left renal calyces. The patient underwent laser ureterorenolithotripsy using a flexible device, followed by extracorporeal shock wave lithotripsy (ESWL) approximately six months later. These interventions were not entirely successful, as significant amounts of calcified debris persisted. Approximately four months after the ESWL, after attending a physiotherapy session for osteopathic treatment, the patient reported the spontaneous passage of multiple residual stones during urination. The patient noted a correlation between the right lateral decubitus position and expulsion of stone fragments. To test his hypothesis, he adopted this position once a day for 40 minutes, immediately followed by the Credé maneuver to void the bladder. After each session, multiple residual stones were expelled. By the third day, no further stones were eliminated, and follow-up X-rays showed no remaining fragments. The findings of this case report may contribute to improving kidney stone elimination in patients with spinal cord injuries. A possible mechanism for the clearance of residual stones in the left calyceal region while in the right lateral decubitus position is that this posture creates a more direct pathway for fragment expulsion, optimizing the effects of gravity and urinary flow.

## Introduction

Individuals with neurogenic dysfunction of the urinary system due to spinal cord injury (SCI) are at higher risk of developing renal calculi [1]. The incidence of renal calculi in individuals with SCI can be up to six times higher than in the general population [2]. It is more frequent in males than in females, with a ratio of approximately 2:1 [1], and the peak of occurrence occurs between three and nine months after SCI [3]. Individuals with renal calculi are more susceptible to infections and sepsis, which can be a life-threatening situation [4]. The immobilization of the lower limbs after SCI injury leads to increased osteoclastic activity, resulting in hypercalcemia and hypercalciuria, which are believed to be among the primary factors in the development of calculi in SCI patients [5].

The treatment of renal calculi can be performed by several methods. The most common are surgery for large stones and stone fragmentation using extracorporeal lithotripsy or ureteroscopy with laser lithotripsy [6,7]. Despite recent advances in patient care and technology, the clearance of residual fragments after lithotripsy remains a problem of clinical relevance [8]. Residual fragments are more frequently observed in renal calyces and the lower renal pelvis [9,10]. Recurrence rates after lithotripsy range from 34% to 78%, depending on the period after intervention [9,11]. Individuals with SCI frequently present some degree of renal dysfunction, which can be aggravated by the presence of calculi requiring removal [12].

This case report describes a possible protocol to facilitate the clearance of large amounts of residual calcified fragments from the kidney in patients with paraplegia of the lower limbs.

## Case presentation

**Figure 1:**
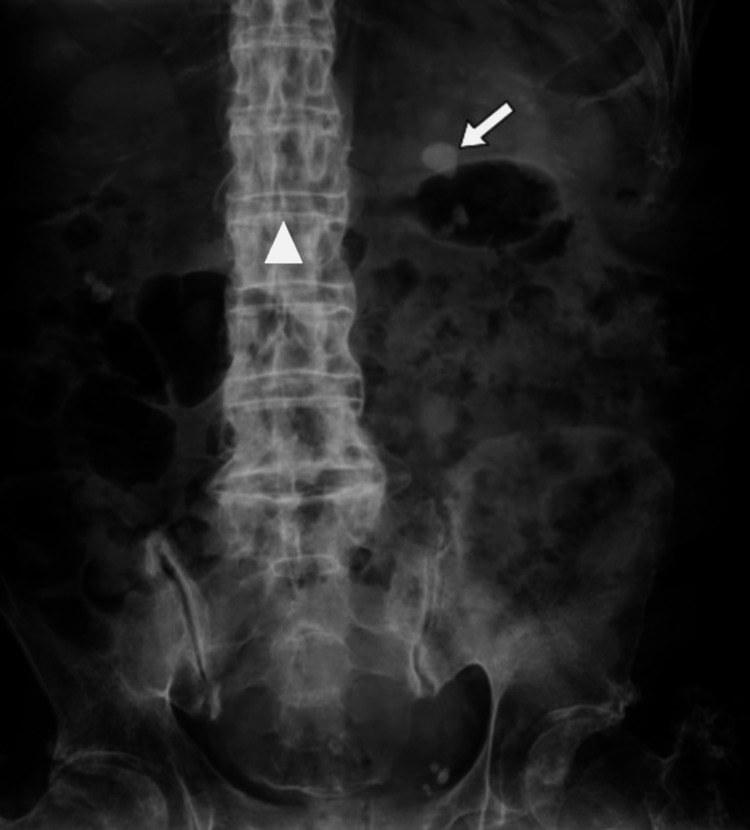
Preoperative X-ray showing a kidney stone in the lower pole of the left kidney (arrow). The spine is indicated by the arrowhead.

**Figure 2:**
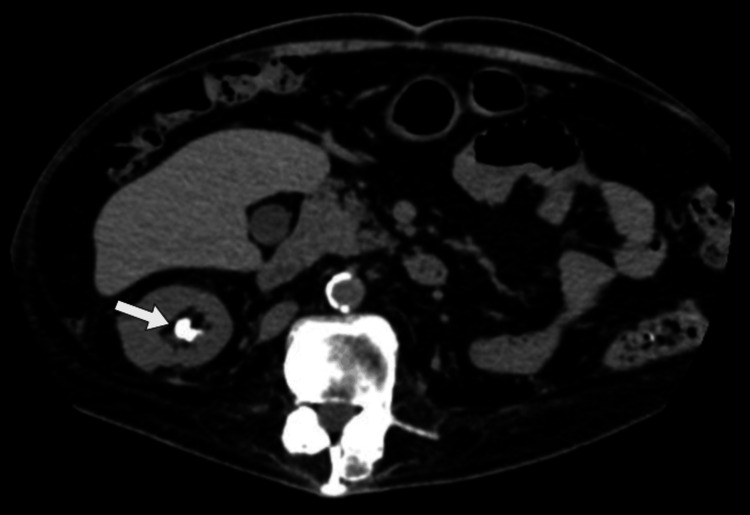
Preoperative magnetic resonance imaging showing stones in the pelvic and caliceal regions of the lower left kidney (arrow).

The patient was a 63-year-old male at the time of the stone fragmentation procedure. He had sustained an SCI at the age of 16 due to an abscess in the thoracic region. This resulted in complete paraplegia of the lower limbs. A preoperative X-ray revealed two kidney stones in near proximity in the lower left renal calyces (Figure 1). They were located in the lower left kidney, measuring 17 and 15 mm (Figure 2). The X-ray was taken during a routine annual check-up with the urologist. The patient was asymptomatic concerning the renal calculus.

**Figure 3:**
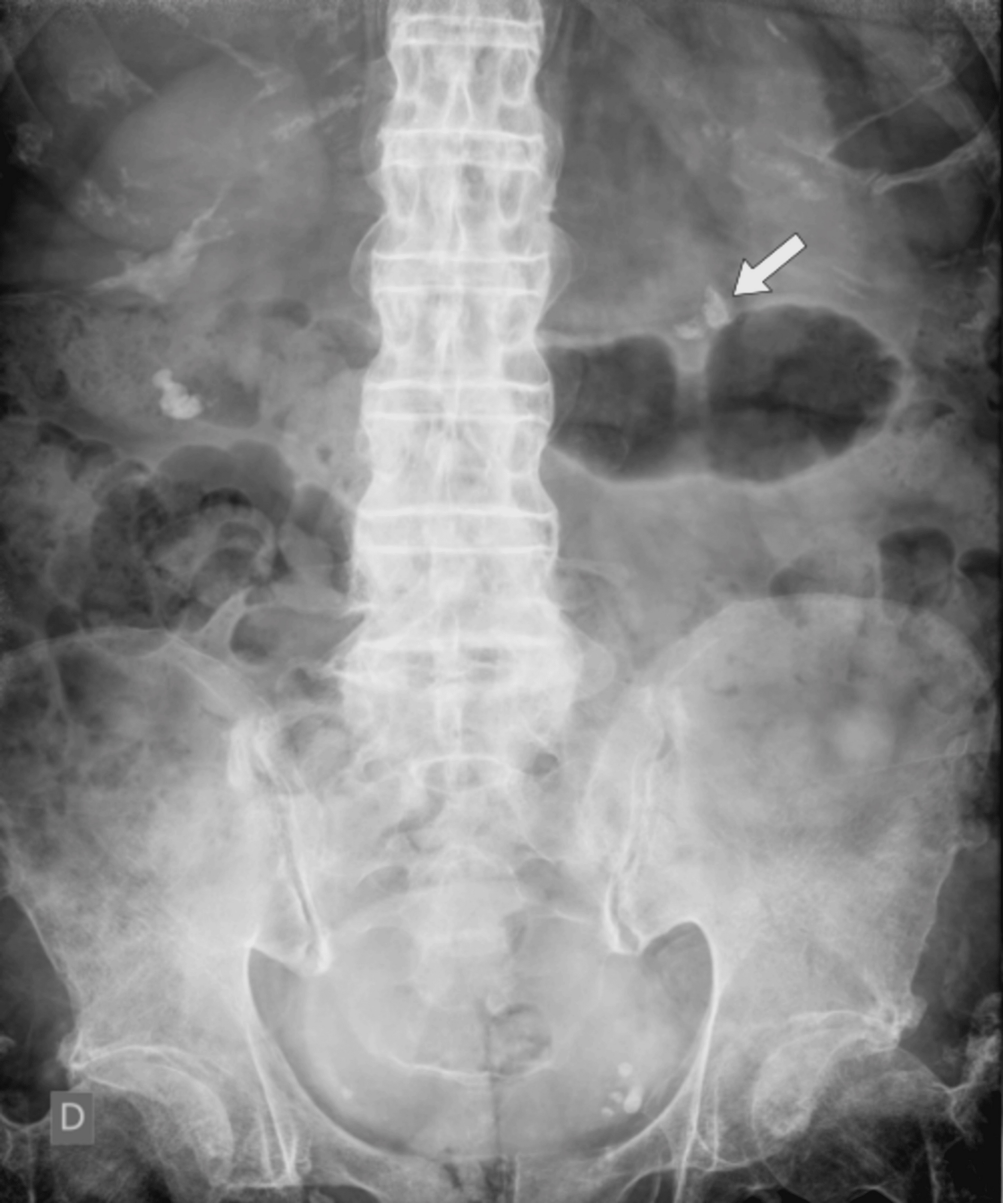
Postoperative X-ray (laser ureterorenolithotripsy with a flexible device) showing radiopaque granular masses with irregular borders located near the original position of the stones (arrow).

In April 2023, the patient underwent laser ureterorenolithotripsy using a flexible device. A postoperative X-ray showed radiopaque, granular masses with irregular borders near the original location of the stones (Figure 3), suggesting residual fragments.

**Figure 4:**
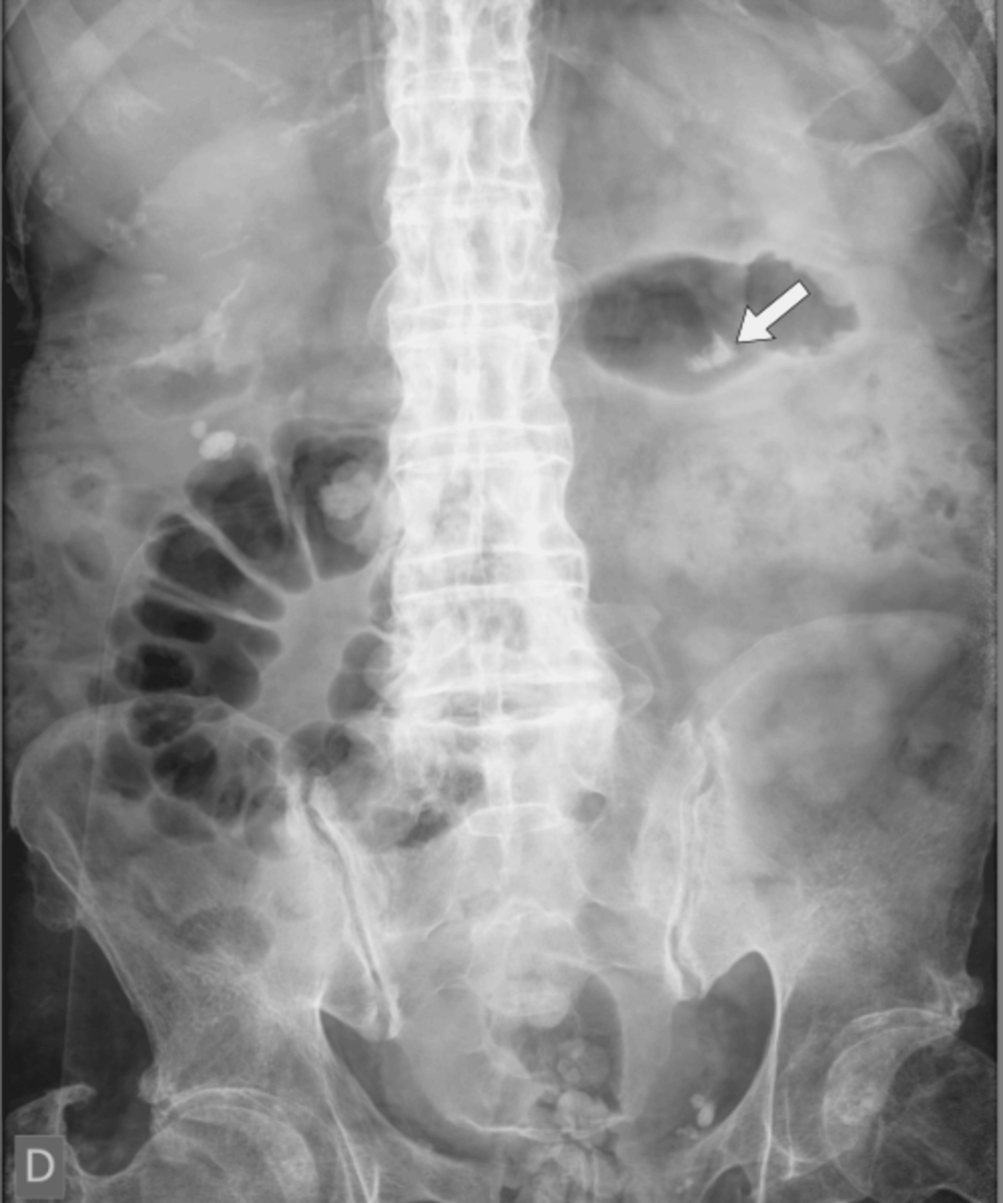
An X-ray performed after extracorporeal shock wave lithotripsy. Note the presence of residual stones (arrow).

Therefore, the patient underwent extracorporeal shock wave lithotripsy (ESWL) in September 2023, 141 days after ureterorenolithotripsy. The treatment was ineffective, as no significant changes were observed in the X-ray patterns (Figure 4).

These findings suggest that, despite the fragmentation of the original stones, a substantial portion of the calcified material remained and was not expelled through urine.

In January 2024, 267 days after laser ureterorenolithotripsy and 127 days after ESWL, the patient attended an assessment physiotherapy session for osteopathy, which was unrelated to the kidney stones. There was no manipulation of the patient during the assessment section. During the session, the patient remained in the right lateral decubitus position. After the session, while urinating, he expelled a significant amount of residual stones. Notably, he insightfully associated the lateral decubitus position with the elimination of these stones. It is also worth mentioning that the patient is a university professor and researcher in the health field, with extensive knowledge of scientific methodology and hypothesis testing.

**Figure 5:**
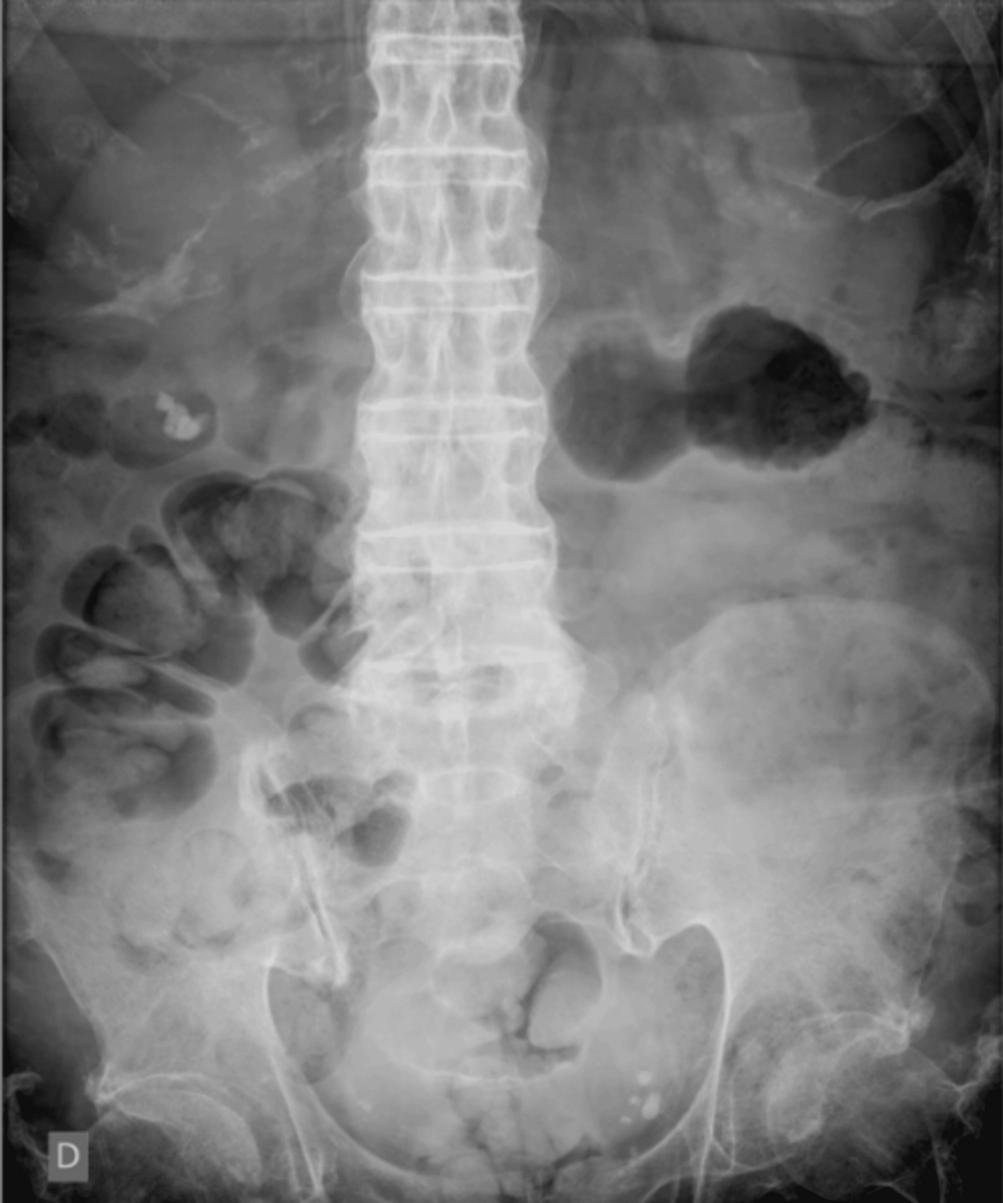
X-ray performed after four procedures of right lateral decubitus. Note the absence of calcified material in the left kidney.

**Figure 6:**
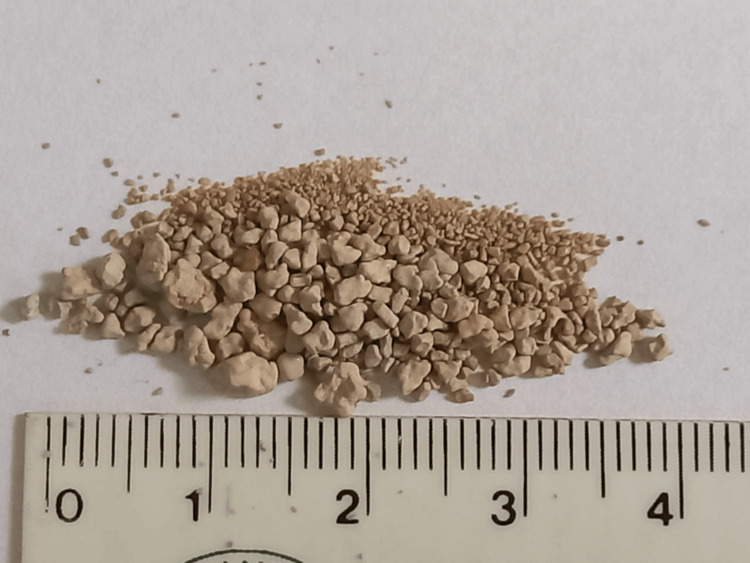
Residual stones collected after three sections of right lateral decubitus followed by the Credé maneuver.

To test his hypothesis, the patient assumed the right lateral decubitus position once daily for approximately 40 minutes, followed immediately by the Credé maneuver [13]. The maneuver is a manual medical technique used to help empty the urinary bladder in patients who have lost the ability to urinate. It involves mechanical compression of the bladder to increase intravesical pressure and force urine out through the urethra. To his surprise, numerous residual stones were expelled after this process. Notably, the patient maintained a routine of four daily urination sessions using the Credé maneuver, yet stone expulsion occurred only when he was positioned in the right lateral decubitus position. Importantly, he was able to assume this position independently without assistance. This procedure was repeated for three consecutive days until no further residual calculi were observed. X-ray imaging confirmed the absence of residual stones in the left kidney (Figure 5), and the expelled fragments from all three sessions are shown in Figure 6.

## Discussion

Complete clearance of residual fragments can have a significant impact on the health of SCI patients and on reducing healthcare costs. Due to the relevance of this subject matter, several therapies have been developed to improve the clearance of residual fragments [12]. An 11% increase in clearance of calyceal stones was achieved by maintaining a 30-degree head-down inclination in patients with lower caliceal stones during ESWL [14]. The use of external physical vibration lithecbole after ESWL significantly increased the time and quantity of residual stones released during the first two weeks after treatment [15]. More recently, it has been shown that kidney stones smaller than 5 mm can be displaced by ultrasound propulsion bursts [16-18]. These therapies, however, require the use of electronic medical apparatuses, specialized personnel, and patients' visits to treatment site centers. This, in turn, renders the therapy more expensive and laborious.

**Figure 7:**
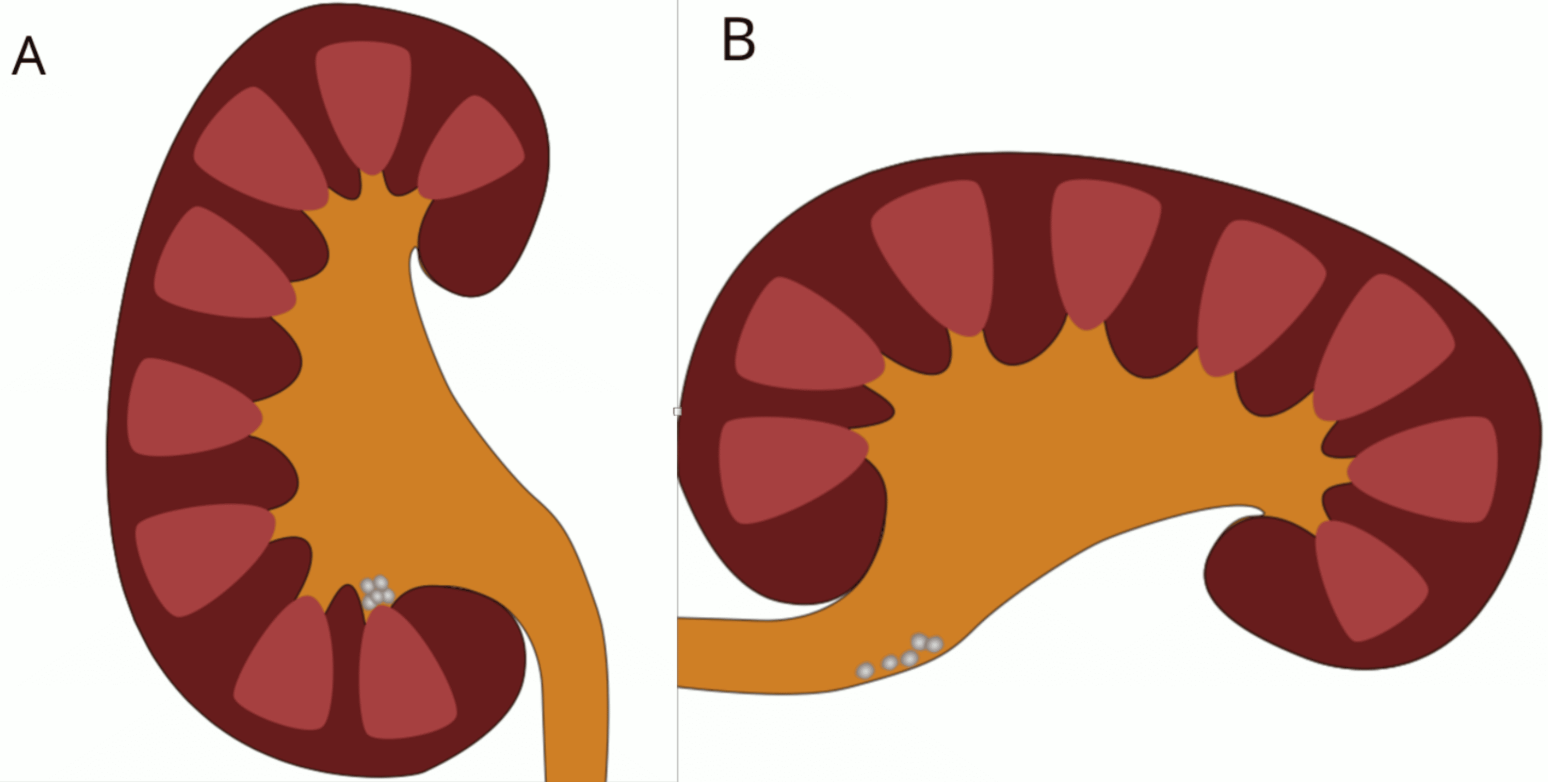
Possible mechanism explaining the clearance of residual stones in the left lower renal calyces while in the right lateral decubitus position. (A) Kidney and stones when patients are in an upright, seated, or supine position. (B) Kidney and stones in the right decubitus position. Note that this position facilitates the action of the force of gravity upon fragments. Image credits: The authors

The impact of anatomical factors is well demonstrated in studies examining the influence of lower-pole kidney anatomy on fragment clearance [19]. These authors analyzed stone fragment clearance in 74 patients and found that 75% of those with a lower infundibulum-pelvic angle greater than 90° achieved stone-free status within three months. In contrast, only 23% of patients with an angle smaller than 90° were stone-free in the same period. These findings underscore the significant role of anatomical variations in determining treatment outcomes. Lower-pole kidney stones are not only more common but also exhibit the lowest success rate of clearance following lithotripsy. This phenomenon is likely attributed to the unique anatomical features of this region. The presence of long calyceal necks, narrow infundibulocalyceal angles, and a narrow infundibulum contributes to the immobilization of stone fragments within these structures. Additionally, the gravity-dependent position of the lower pole further hinders the clearance of stones or fragments. In seated, upright, or supine positions, these anatomical characteristics are positioned lower than the ureter and create an anatomical depression, resulting in a gravity-unfavorable environment for the passage of fragments (Figure 7A). The right lateral decubitus position creates a more favorable topography for the displacement of the residual fragments in the lower pole of the left kidney (Figure 7B). Gravitational-dependent anatomical aspects significantly influence the stone-free rate after renal stone fragmentation [20].

## Conclusions

The findings of this case report suggest a method to facilitate kidney stone elimination in patients with SCI. A possible mechanism for the clearance of residual stones from the left calyceal region, specifically while the patient is in the right lateral decubitus position, involves the optimization of gravitational forces. It is worth noting that successful fragment clearance was achieved after four sessions; however, this outcome may vary among patients. Significantly, the stone removal protocol was developed empirically based on patient observations and is designed to be performed independently by the patient, without requiring third-party assistance.
